# A population-based predictive model identifying optimal candidates for primary and metastasis resection in patients with colorectal cancer with liver metastatic

**DOI:** 10.3389/fonc.2022.899659

**Published:** 2022-10-07

**Authors:** Xin Jin, Yibin Wu, Yun Feng, Zhenhai Lin, Ning Zhang, Bingran Yu, Anrong Mao, Ti Zhang, Weiping Zhu, Lu Wang

**Affiliations:** Department of Hepatic Surgery, Fudan University Shanghai Cancer Center, Shanghai Medical College, Fudan University, Shanghai, China

**Keywords:** stage M1a colorectal cancer, liver metastases, resection of primary and metastatic lesions, SEER database, nomogram

## Abstract

**Background:**

The survival benefit of primary and metastatic resection for patients with colorectal cancer with liver metastasis (CRLM) has been observed, but methods for discriminating which individuals would benefit from surgery have been poorly defined. Herein, a predictive model was developed to stratify patients into sub-population based on their response to surgery.

**Methods:**

We assessed the survival benefits for adults diagnosed with colorectal liver metastasis by comparing patients with curative surgery vs. those without surgery. CRLM patients enrolled in the Surveillance, Epidemiology, and End Results (SEER) database between 2004 and 2015 were identified for model construction. Other data including CRLM patients from our center were obtained for external validation. Calibration plots, the area under the curve (AUC), and decision curve analysis (DCA) were used to evaluate the performance of the nomogram compared with the tumor–node–metastasis (TNM) classification. The Kaplan–Meier analysis was performed to examine whether this model would distinguish patients who could benefit from surgery.

**Results:**

A total of 1,220 eligible patients were identified, and 881 (72.2%) underwent colorectal and liver resection. Cancer-specific survival (CSS) for the surgery group was significantly better than that for the no-surgery group (41 vs. 14 months, p < 0.001). Five factors were found associated with CSS and adopted to build the nomograms, i.e., age, T stage, N stage, neoadjuvant chemotherapy, and primary tumor position. The AUC of the CRLM nomogram showed a better performance in identifying patients who could obtain benefits in the surgical treatment, compared with TNM classification (training set, 0.826 [95% CI, 0.786–0.866] vs. 0.649 [95% CI, 0.598–0.701]; internal validation set, 0.820 [95% CI, 0.741–0.899] vs. 0.635 [95% CI, 0.539–0.731]; external validation set, 0.763 [95% CI, 0.691–0.836] vs. 0.626 [95% CI, 0.542–0.710]). The calibration curves revealed excellent agreement between the predicted and actual survival outcomes. The DCA showed that the nomogram exhibited more clinical benefits than the TNM staging system. The beneficial and surgery group survived longer significantly than the non-beneficial and surgery group (HR = 0.21, 95% CI, 0.17–0.27, p < 0.001), but no difference was observed between the non-beneficial and surgery and non-surgery groups (HR = 0.89, 95% CI, 0.71–1.13, p = 0.344).

**Conclusions:**

An accurate and easy-to-use CRLM nomogram has been developed and can be applied to identify optimal candidates for the resection of primary and metastatic lesions among CRLM patients.

## Background

Colorectal cancer (CRC) is the third most common cancer worldwide, and 50% of patients develop liver metastasis during the course of the disease (1, 2). Among the potential curative therapies, primary and metastatic resection are the primary option to improve the prognosis of patients (3, 4). However, there remains substantial heterogeneity for some patients with resectable colorectal cancer with liver metastasis (CRLM). At present, the tumor–node–metastasis (TNM) staging classification is mainly considered in the prediction of CRLM prognosis (5). However, some studies demonstrates that CRLM patients with the same TNM classification have a different clinical outcome, and many valuable clinical factors are neglected, which are associated with the prognosis of CRLM patients undergoing primary and metastatic resection (6, 7).

More precise categorization is needed to identify those who may benefit more from surgery. Thus, it is necessary to stratify patients based on their preoperative features to provide more individualized treatments. All kinds of prediction models have been developed and validated to overcome the drawback of the TNM classification system (8). Among these models, the nomogram developed based on several independent prediction factors was widely considered an accurate and easy-to-use tool to visualize the prognosis of patients individually (9–12). It has been reported that the C-index of the nomogram predicting the risk of bone metastasis in colorectal cancer reached 0.929 (13).

Although some studies (14–16) have explored the nomogram to predict the prognosis of CRLM patients, they only predicted the overall survival, and they did not inform patients if they could live longer without the surgery. In this study, a new clinical outcome was established, which included a comparison with the median survival time of non-surgical patients. We assumed that patients receiving surgical treatment who lived longer than the median cancer-specific survival (CSS) time of those who did not undergo surgery could benefit from the operation. Based on this unusual clinical outcome, we aimed to investigate the preoperative prognostic factors, develop and validate an effective predictive model, and then make a reference standard based on the possibility of benefit to identify CRLM patients who would benefit from resection of primary and metastatic lesions.

## Method

### Patient

For this study, the data we analyzed were extracted from the Surveillance, Epidemiology, and End Results (SEER) database (2000–2018, November 2020 submission), which covers approximately 28% of the US population (17). The SEER*Stat, Version 8.3.9, was applied to examine the data for research between 2004 and 2015.

The inclusion criteria for patients were as follows: 1) patients came from the database of “Incidence—SEER Research Plus Data, 18 Registries, Nov 2020 Sub (2000-2018)”. 2) The International Classification of Diseases for Oncology (ICD-O-3) was used for the CRLM definition. “Site recode ICD-O-3/WHO 2008” was used to record tumor location information, including ascending colon, hepatic flexure, transverse colon, splenic flexure, descending colon, sigmoid colon, rectosigmoid junction, and cecum. 3) Liver metastasis. 4) Single primary site. 5) The TNM stage was stated as “M1a”. “M1a” was confined as “Metastasis limited to a single distant organ except for peritoneum” (6), and “Year of diagnosis” was set to 2004–2015. 7) According to “Histologic Type ICD-O-3”, the following pathological types were included in the following: adenocarcinoma (8140), adenocarcinoma arising in a polyp (8210), adenocarcinoma in tubulovillous adenoma (8263), mucinous/colloid adenocarcinoma (8480), and adenosquamous carcinoma (8560).

The exclusion criteria were as follows: 1) less than 20 years old; 2) diagnosed with no positive histology and not only from a death certificate or autopsy; 3) the information about the surgery to the primary site and metastatic lesion was missing; 4) clinical pathological information (tumor size, carcinoembryonic antigen (CEA), T stage, N stage, histologic type, and neoadjuvant chemotherapy) was missing; and 5) survival information (survival month and final cause of death) was missing.

CRLM patients treated in the Shanghai Cancer Center, Fudan University (FUSCC) from 2016 to 2017 were enrolled as an independent external validation set for this study. The included criteria are as follows: 1) over 18 years old; 2) single primary site; 3) diagnosed with positive histology; 4) the TNM stage was stated as “M1a”; and 4) complete demographic data, clinical parameters, TNM stage information, and full follow-up results.

### Data collection

The analyzed data included age (<50, 50 ≤ X < 70, and ≥70), sex (male and female), primary site (rectum, and left and right colon), tumor size (>5 and ≤5 cm), CEA (positive and negative/normal), T stage (T1, T2, T3, and T4), N stage (N0, N1, and N2), differentiation grade (Grade I, Grade II, Grade III, Grade IV, and unknown), histologic type (adenocarcinoma and others), neoadjuvant chemotherapy (yes and no), marital status (married, separated/divorced, single, widowed, and unknown), surgery to the primary site (yes and no), and surgery to the metastatic lesions (yes and no). Overall survival (OS), CSS, and survival month were extracted from the SEER database. OS time was defined as the time from diagnosis to death or to the time of data analysis. Living patients were excluded at the time of the last recording. The CSS duration can be calculated from the date of diagnosis to a documented CRC-related death. According to the published papers (18), we identified “Nonprimary surgical procedure to distant site” as the resection of metastatic lesions.

### Statistical analysis

The research group was separated into two groups based on therapy, surgery versus non-surgery. Clinical differences (categorical variables) were represented as a number with percentage and compared by using the chi-square test and Fisher’s exact test. The Kaplan–Meier (K-M) method and the log-rank test were analyzed in two groups to confirm the influence of surgery on the survival of patients. To identify independent predictors, univariate and multivariate Cox proportional hazards regression analyses were performed. Hazard ratios (HRs) were calculated with 95% confidence intervals (CIs). Statistical data were analyzed with SPSS 24.0 (IBM Corp, Armonk, NY, USA). All statistical tests were two-sided, and only p < 0.05 could be regarded as statistically significant.

### Construction and validation of the nomogram

The eligible people receiving surgery were randomly divided into the training and validation cohorts. Patients who survived longer than the median CSS time of those who received no surgery were defined as benefiting from the surgical treatment. According to the univariate and multivariate Cox analyses, the factors independently affecting the CSS were indicated in the training cohort. Based on the multivariable logistic analysis, the nomogram to identify the patients who may obtain benefits from primary and metastasis resection was developed. The areas under the receiver operating characteristic (ROC) curve (AUCs) were applied to quantify ROC performance to assess the discriminative and calibration capacity of the nomograms. Calibration curves were utilized to demonstrate no deviations from the reference line, indicating a high degree of dependability. What is more, the decision curve analysis (DCA) was also used to evaluate the clinical application value and clinical practicability of the models. Overall, we used ROC, calibration plots, and DCA to graphically describe the performance of our model. What is more, we attempted to assign all CRLM patients undergoing surgery to two groups—beneficial and surgery group and non-beneficial and surgery group—in terms of probability of benefit of 50%. The Kaplan–Meier analyses and the log-rank test were employed to test whether this model could identify individuals who could indeed benefit from the resection of primary and metastatic lesions.

## Results

### Patient characteristics

**Figure 1:**
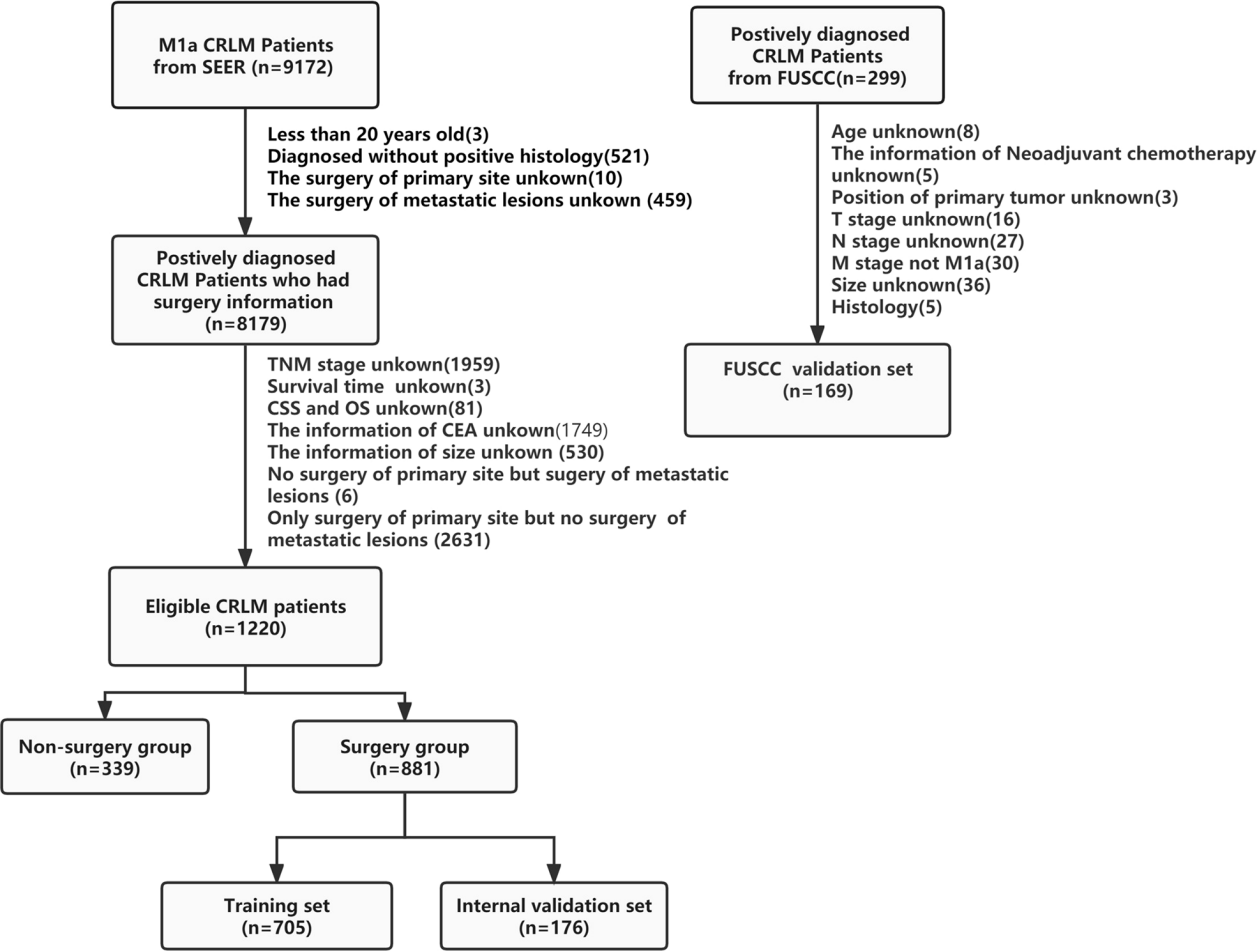
Flowchart of the data selection process.

From the SEER database, 1,220 patients with M1a CRLM who met inclusion criteria were identified from 2004 to 2015. The flowchart is illustrated in **Figure 1**. Among them, 881 (72.2%) received primary and metastatic resection, while 339 (27.8%) had no surgical treatment. Men predominantly made up 53.4% of these cases, 20.7% of these patients were under the age of 50, and 15.1% of the tumors were in the rectum; 66.2% of tumors were classified as grade II in terms of the differentiation grade. In addition, 80.6% of patients were CEA-positive. Adenocarcinoma was found in 87.0% of the patients. Also, neoadjuvant chemotherapy was administered to 81.3% of patients. According to the TNM stage classification, 54.3% of the tumor were categorized as T3, and 27.8% of them were categorized as N2. The detailed clinical information of all patients is summarized in **Table S1**.

### Least absolute shrinkage and selection operator regression

**Figure 2:**
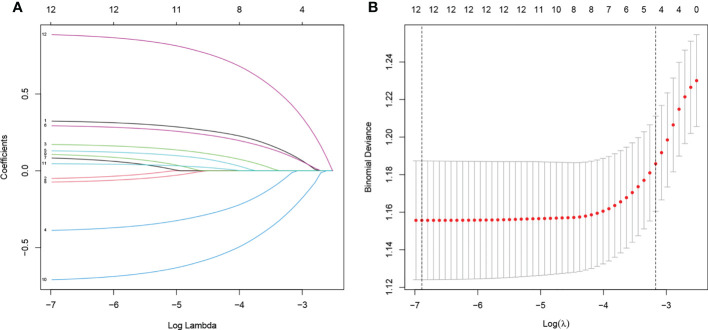
**(A)** Plot of partial likelihood deviance of CSS. **(B)** LASSO coefficient profile plot of CSS. CSS, cancer-specific survival; LASSO, least absolute shrinkage and selection operator.

In total, 12 variables were incorporated in the least absolute shrinkage and selection operator (LASSO) regression, and all of them were included: age, sex, race, differentiation grade, histology type, T stage, N stage, tumor size, marital status, neoadjuvant chemotherapy, CEA, and primary tumor position (**Figure 2**).

### Kaplan–Meier curves of cancer-specific survival

According to the K-M analysis and log-rank test in the SEER cohort (**Figure S1**), patients who received excision of primary and metastatic tumors enjoyed a longer CSS. The median CSS time was 41 months (95% CI, 37.15–44.85) for individuals who underwent resection of primary and metastatic tumors, compared to 14 months (95% CI, 11.37–16.63) for patients with no surgery.

### Univariable and multivariable analyses

Compared with logistic regression, the Cox analysis focused more on the influence of variables on the survival of CRLM patients. In the univariate and multivariate Cox analyses, age, race, differentiation grade, primary site, T stage, N stage, neoadjuvant chemotherapy, marital status, and CEA were found to be independent predictors for the survival of patients with stage M1a CRLM. However, sex, tumor size, and histology were shown to have no significant impact on CSS (**Table 1**). Moreover, surgery was found to be independently linked with higher CSS (HR = 0.28, 95% CI, 0.24–0.33, p < 0.001), which further indicated the significance of surgery in the treatment.

**Table 1:** Univariable and multivariate Cox analyses for CSS among CRLM patients.

	Univariable		Multivariable	
	Adjust HR (95% CI)	p-Value	Adjust HR (95% CI)	p-Value
Age				
<50	References		References	
50 ≤ X < 70	1.34 (1.12–1.62)	0.001	1.14 (0.95–1.38)	0.16
≥70	2.67 (2.17–3.27)	<0.001	1.53 (1.21–1.93)	<0.001
Sex				
Female	References			
Male	0.93 (0.81–1.06)	0.274		
Size				
≤3 cm	References		References	
3 < X ≤ 5 cm	0.88 (0.72–1.07)	0.204	0.84 (0.69–1.03)	0.1
5 < X ≤7 cm	1.07 (0.87–1.32)	0.534	0.95 (0.76–1.18)	0.63
>7 cm	1.45 (1.16–1.83)	0.001	1.19 (0.93–1.51)	0.17
Race				
Black	References		References	
White	0.82 (0.69–0.98)	0.029	0.84 (0.70–1.01)	0.062
Other	0.61 (0.46–0.82)	<0.001	0.60 (0.45–0.81)	0.001
Grade				
I	References		References	
II	0.87 (0.59–1.29)	0.48486	0.96 (0.65–1.44)	0.852
III	1.51 (1.00–2.29)	0.05247	1.57 (1.03–2.40)	0.04
IV	1.48 (0.88–2.46)	0.13615	1.86 (1.10–3.14)	0.02
Unknown	1.93 (1.27–2.95)	0.002	1.17 (0.75–1.81)	0.5
Histology				
Other	References			
Adenocarcinoma	1.16 (0.95–1.42)	0.155		
T stage				
T1	References		References	
T2	0.21 (0.13–0.34)	<0.001	0.43 (0.26–0.71)	0.001
T3	0.36 (0.30–0.43)	<0.001	0.75 (0.60–0.95)	0.019
T4	0.57 (0.47–0.70)	<0.001	1.03 (0.81–1.32)	0.785
N stage				
N0	References		References	
N1	0.74 (0.63–0.86)	<0.001	1.08 (0.91–1.29)	0.377
N2	0.82 (0.69–0.97)	0.021	1.45 (1.18–1.80)	<0.001
Neoadjuvant chemotherapy				
No	References		References	
Yes	0.27 (0.23–0.31)	<0.001	0.29 (0.24–0.34)	<0.001
Marital status				
Married	References		References	
Separated or divorced	1.23 (0.99–1.52)	0.067	0.92 (0.74–1.15)	0.478
Single	1.28 (1.07–1.52)	0.006	1.21 (1.01–1.45)	0.04
Widowed	2.10 (1.68–2.61)	<0.001	1.22 (0.96–1.55)	0.1
Unknown	1.28 (0.87–1.88)	0.206	1.10 (0.74–1.63)	0.633
CEA				
Negative/normal	References		References	
Positive/elevated	1.89 (1.56–2.28)	<0.001	1.86 (1.53–2.26)	<0.001
Primary tumor position				
Left colon	References		References	
Right colon	1.63 (1.41–1.89)	<0.001	1.49 (1.28–1.74)	<0.001
Rectum	1.07 (0.87–1.32)	0.517	0.92 (0.74–1.14)	0.438
Surgery				
No	References		References	
Yes	0.28 (0.24–0.33)	<0.001	0.30 (0.25–0.38)	<0.001

CRLM, colorectal cancer with liver metastasis; CEA, carcinoembryonic antigen; HR, hazard ratio; CI, confidence interval.

p < 0.05 means the result is statistically significant.

### Definition of benefiting in the surgery

The median CSS time (14 months) of non-surgical patients was considered as the reference line. Patients who underwent curative surgery yielded better CSS than this reference line and were identified to be beneficial in the operation. Conversely, surgical patients whose CSS was lower than 14 months were considered non-beneficial patients. A total of 708 (80.36%) patients were categorized as “beneficial”. The characteristics of patients are presented in **Table 2**.

**Table 2:** Characteristics of M1a CRLM patients who benefit from the surgery.

Parameters	All surgery M1a CRLM patientsn = 881 (%)	Non-beneficial n = 173 (%)	Beneficial n = 708 (%)	p-Value
Age				<0.001
<50	214 (24.3)	17 (9.8)	197 (27.8)	
50 ≤ X < 70	483 (54.8)	77 (44.5)	406 (57.4)	
≥70	184 (20.9)	79 (45.7)	105 (14.8)	
Sex				0.099
Female	414 (47.0)	91 (52.6)	323 (45.6)	
Male	467 (53.0)	82 (47.4)	385 (54.4)	
Size				0.007
≤3 cm	146 (16.6)	22 (12.7)	124 (17.5)	
3 < X ≤ 5 cm	376 (42.7)	68 (39.3)	308 (43.5)	
5 < X ≤ 7 cm	227 (25.7)	43 (24.9)	184 (26.0)	
>7 cm	132 (15.0)	40 (23.1)	92 (13.0)	
Race				0.544
Black	135 (15.3)	21 (12.2)	114 (16.1)	
White	654 (74.2)	135 (78.0)	519 (73.3)	
Other	91 (10.4)	17 (9.8)	74 (10.5)	
Unknown	1 (0.1)	0 (0.0)	1 (0.1)	
Grade				<0.001
I	26 (3.0)	4 (2.3)	22 (3.1)	
II	646 (73.3)	102 (59.0)	544 (76.8)	
III	140 (15.9)	51 (29.5)	89 (12.6)	
IV	38 (4.3)	13 (7.5)	25 (3.5)	
Unknown	31 (3.5)	3 (1.7)	28 (4.0)	
Histology				0.05
Adenocarcinoma	753 (85.5)	156 (90.2)	597 (84.3)	
Other	128 (14.5)	17 (9.8)	111 (15.7)	
T stage				<0.001
T1	20 (2.3)	3 (1.7)	17 (2.4)	
T2	34 (3.8)	2 (1.2)	32 (4.5)	
T3	582 (66.1)	97 (56.1)	485 (68.5)	
T4	245 (27.8)	71 (41.0)	174 (24.6)	
N stage				<0.001
N0	167 (19.0)	17 (9.8)	150 (21.2)	
N1	390 (44.3)	68 (39.3)	322 (45.5)	
N2	324 (36.8)	88 (50.9)	236 (33.3)	
Neoadjuvant chemotherapy				<0.001
No	126 (14.3)	75 (43.4)	51 (7.2)	
Yes	755 (85.7)	98 (56.6)	657 (92.8)	
Marital status				<0.001
Married	528 (59.9)	95 (54.9)	433 (61.2)	
Separated or divorced	85 (9.6)	17 (9.8)	68 (9.6)	
Single	169 (19.2)	25 (14.5)	144 (20.3)	
Widowed	72 (8.2)	30 (17.3)	42 (5.9)	
Unknown	27 (3.1)	6 (3.5)	21 (3.0)	
CEA				0.109
Negative/normal	209 (23.7)	33 (19.1)	176 (24.9)	
Positive/elevated	672 (76.3)	140 (80.9)	532 (75.1)	
Primary tumor position				<0.001
Left colon	385 (43.7)	49 (28.3)	336 (47.5)	
Right colon	380 (43.1)	108 (62.4)	272 (38.4)	
Rectum	116 (13.2)	16 (9.3)	100 (14.1)	

CRLM, colorectal cancer with liver metastasis; CEA, carcinoembryonic antigen.

p < 0.05 means the result is statically significant.

### Construction and internal validation of the nomogram

We randomly assigned 881 patients who underwent curative surgery in a 4:1 ratio to the training cohort (n = 705) and the validation cohort (n = 176). The median CSS time of the training cohort and validation cohort was 41 [36.74–45.26] and 40 [31.34–48.66] months, respectively.

**Figure 3:**
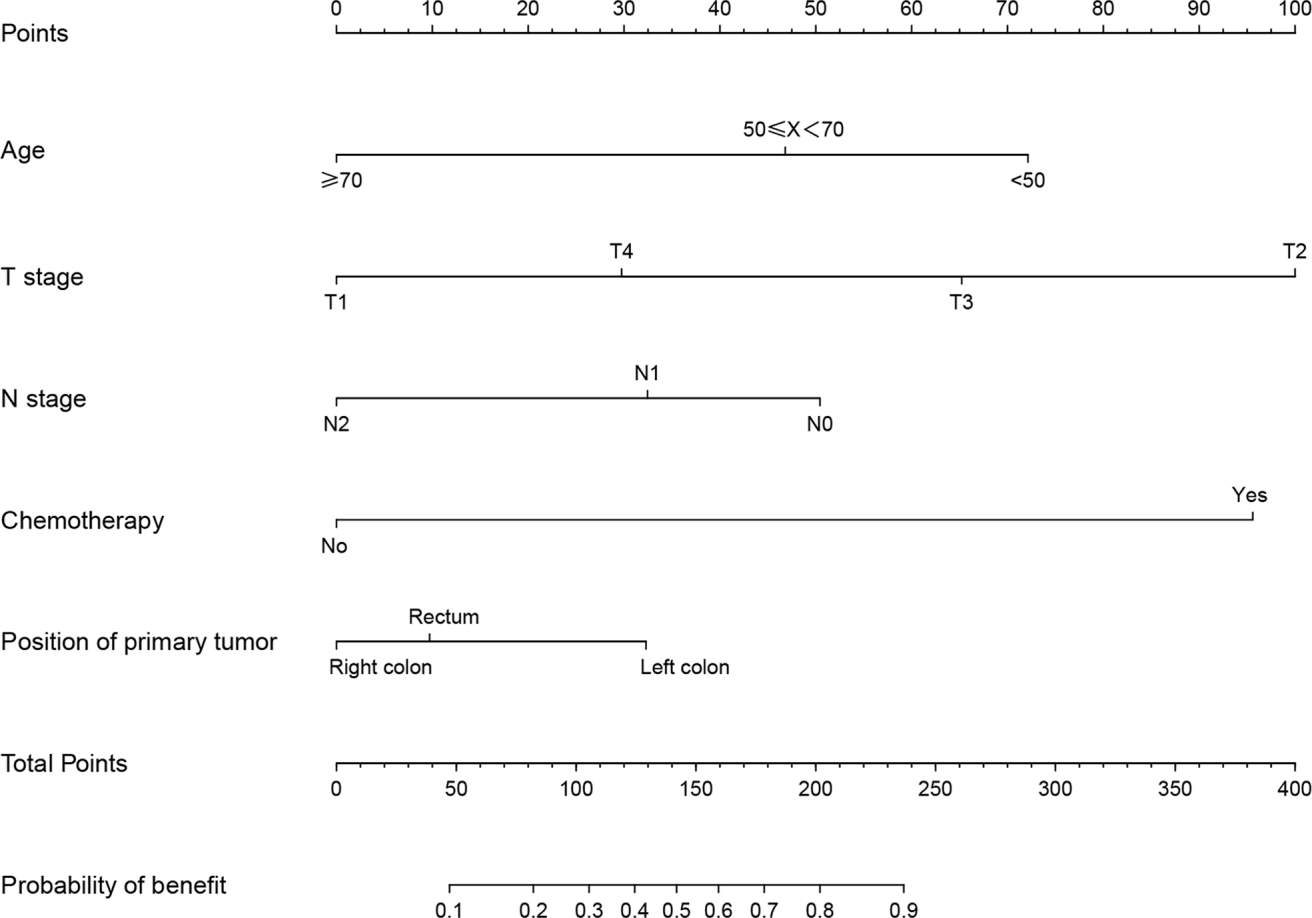
Construction of the CRLM nomogram. CRLM, colorectal cancer with liver metastasis.

Nine independent predictors including age, race, differentiation grade, T stage, N stage, neoadjuvant chemotherapy, marital status, CEA, and primary tumor position were collected into the multivariate logistic regression. We identified five effective factors and developed a nomogram to predict the stage M1a CRLM patients who could benefit from the surgical treatment based on the training cohort (**Figure 3**; **Table 3**).

**Table 3:** Multivariate logistic analysis among M1a CRLM patients.

	Multivariate	
	OR (95% CI)	p-Value
Age		
<50	Reference	
50 ≤ X < 70	0.57 (0.29–1.13)	0.109
≥70	0.21 (0.10–0.45)	<0.001
Race		
Black	Reference	
White	0.63 (0.30–1.31)	0.217
Other	0.60 (0.22–1.60)	0.305
Grade		
I	Reference	
II	1.02 (0.29–3.60)	0.971
III	0.46 (0.12–1.75)	0.255
IV	0.54 (0.12–2.45)	0.423
Unknown	1.64 (0.26–10.27)	0.598
T stage		
T1	Reference	
T2	9.99 (1.23–80.97)	0.031
T3	5.14 (1.20–22.07)	0.028
T4	2.46 (0.56–10.78)	0.234
N stage		
N0	Reference	
N1	0.76 (0.37–1.53)	0.439
N2	0.37 (0.18–0.76)	0.007
Neoadjuvant chemotherapy		
No	Reference	
Yes	9.30 (5.22–16.59)	<0.001
Marital status		
Married	Reference	
Separated or divorced	0.93 (0.43–2.02)	0.852
Single	1.07 (0.56–2.04)	0.836
Widowed	0.77 (0.36–1.63)	0.491
Unknown	0.81 (0.21–3.07)	0.756
CEA		
Negative/normal	Reference	
Positive/elevated	0.57 (0.32–1.02)	0.059
Primary tumor position		
Left colon	Reference	
Right colon	0.52 (0.31–0.87)	0.013
Rectum	0.63 (0.30–1.34)	0.23

CRLM, colorectal cancer with liver metastasis; CEA, carcinoembryonic antigen; HR, hazard ratio; CI, confidence interval.

p < 0.05 means the result is statistically significant.

**Figure 4:**
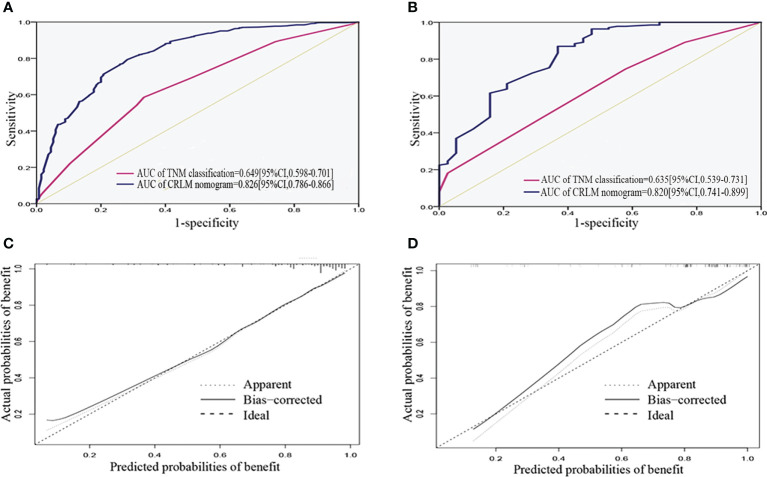
Validation of the nomogram. **(A, B)** ROC curve for discrimination in the training and validation cohorts. **(C, D)** Calibration plots for the actual (observed) and predicted probabilities of the nomograms in the training and validation cohorts. ROC, receiver operating characteristic curve.

**Figure 5:**
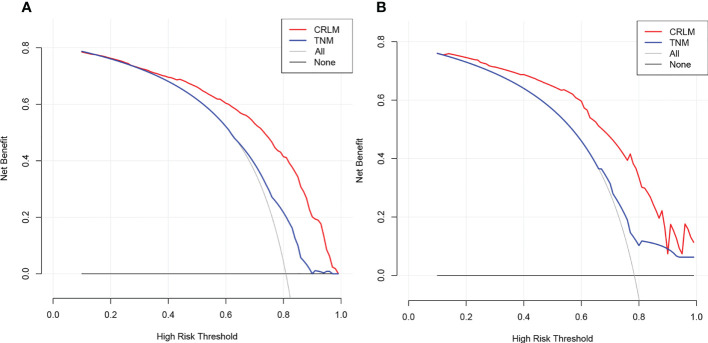
Decision curve analysis of CRLM nomogram in the training **(A)** and validation **(B)** cohorts. Horizontal lines indicate that no cases will experience the event. Gray lines indicate that all cases will experience the event. Red and blue lines represent the net benefits across threshold probabilities according to the CRLM nomogram and TNM classification, respectively. CRLM, colorectal cancer with liver metastasis..

ROC analysis demonstrated the AUCs of the training and validation cohorts reached 0.826 [95% CI, 0.786–0.866] and 0.820 [95% CI, 0.741–0.899], respectively, outperforming the American Joint Committee on Cancer (AJCC)–TNM classification of 0.649 [95% CI, 0.598–0.701] and 0.635 [95% CI, 0.539–0.731], respectively (**Figures 4A, B**). In addition, the performance of the model was visualized by the calibration plots, and the calibration curves showed good agreement between prediction and observation (**Figures 4C, D**). Finally, DCA showed a higher clinical application value and better clinical practicability (**Figure 5**).

### External validation

In this study, external validation was performed in the FUSCC cohort. The AUC of the CRLM nomogram was 0.763 [95% CI, 0.691–0.836], outperforming the AJCC-TNM classification of 0.626 [95% CI, 0.542–0.710] (**Figure S5A**). Moreover, both DCA and the calibration curves of the CRLM nomogram demonstrated better performance compared with the TNM classification (**Figures S5B, C**).

### Development of webserver for easy access to nomogram

According to the above results, a dynamic web-based probability calculator (Dynamic Nomogram (shinyapps.io)) was constructed at https://fusccliver.shinyapps.io/dynnomapp/ to identify optimal candidates for surgery based on the previous nomogram. The predicted probability of benefit in the surgery can be simply calculated by inputting clinical characteristics and viewing the output of the webserver’s output figures and tables.

### Risk stratification system

**Figure 6:**
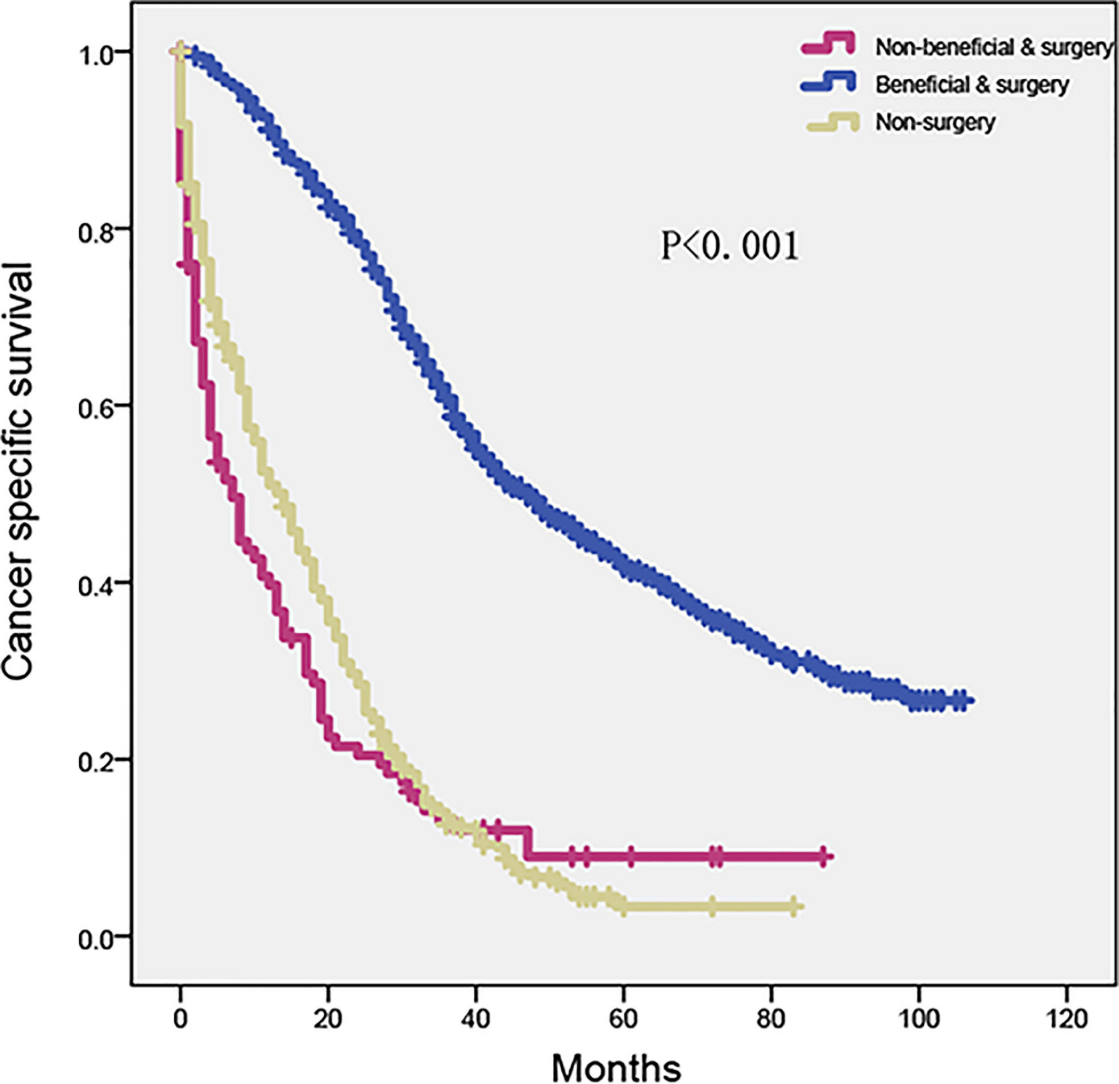
Kaplan–Meier plot to differentiate beneficial group according to CRLM nomogram in SEER cohort. SEER, Surveillance, Epidemiology, and End Results; CRLM, colorectal cancer with liver metastasis.

According to the Kaplan–Meier analysis and log-rank test, the beneficial and surgery group had a considerably longer survival time than the non-beneficial and surgery group (HR = 0.21, 95% CI, 0.17–0.27, p < 0.001). However, no significant difference was found between the non-beneficial and surgery and non-surgery groups (**Figure 6**) (HR = 0.89, 95% CI, 0.71–1.13, p = 0.344).

## Discussion

In the present study, a nomogram including age, T stage, N stage, neoadjuvant chemotherapy, and primary tumor position was constructed and validated to identify optimal candidates for the primary and metastatic resection. In clinical practice, our nomogram can aid clinicians in the process of making decisions as a convenient and accurate predictive model.

Five factors were considered into account in our model in this study and were attached to different risk scores, which could indicate the impact they did on the decision. Present results supported our hypothesis and revealed some significant discoveries. Our nomogram shared several parameters with previous studies on CRLM survival prediction. Some factors marked with a high-risk score in our model, like T stage, age, neoadjuvant chemotherapy, and primary tumor position, were also generally recognized in other studies (12, 15, 16, 19, 20).

For the first time, age was indicated to correlate with the potential of benefit strongly. A growing body of evidence suggests that the elderly have a poorer prognosis, which is consistent with our result (21–23). The elderlies undergoing surgery usually have poor physical and mental health, which has a bad effect on the subsequent adjuvant therapy. What is more, due to neglect of regular physical examination, the tumor is often at an advanced stage when discovered.

In our nomogram, the T1 stage had the least risk scores, indicating that patients with T1-stage tumors are unlikely to benefit from surgery. This distinct phenomenon went against common sense. However, the research of Lupo Wu also noticed this phenomenon and attributed it to the distinct genetic profile of the T1 stage tumors (24). This finding indicated that more attention should be paid to the surveillance and the screening of CRLM with early T stage.

The primary tumor sites served as a high-ranking risk factor, which could affect the potential surgery benefit in our models, and other studies have also confirmed this observation (20, 25, 26). Patients with left-sided tumors often had a better survival outcome than those with right-sided tumors, with a longer CSS of 89 vs. 78 months (p = 0.001) in a SEER cohort (27). Moreover, a national multi-center retrospective study launched by Shida demonstrated that right-sided CRC (RCRC) patients had worse OS than left-sided CRC (LCRC) patients (22). Some studies revealed that histological and molecular characteristics played an important role in this phenomenon (22, 28–30). The gene profile of RCRC and LCRC is completely different. RCRC was mostly diploid with high microsatellite instability, mucinous histology, CpG island methylation, and BRAF mutation, which made RCRC tend to have a more advanced clinical behavior than LCRC. Conversely, LCRC has frequent p53 and KRAS mutations (29, 31). Additionally, it is more difficult to detect RCRC at an early stage because of its flat morphology in the screening of colonoscopy (32, 33). Therefore, the primary lesion of RCRC is often discovered in more advanced stages than that of LCRC.

The tumor size and grade demonstrated a correlation to CSS based on LASSO regression. Nevertheless, the strength of correlation cannot meet the criteria for multiple variable Cox and logistic regression. Therefore, they were excluded from our prediction models. A similar outcome was found in other CRLM studies (12, 15, 16). Conversely, sex was proved that it had a certain effect on the outcome in Kattan’s research (19), and we attributed the cause to the difference between the data from different centers for this conflict.

Additionally, our research demonstrated that carcinoembryonic antigen (CEA) was associated with OS and CSS in LASSO regression. Some studies have indicated that preoperative serum CEA level plays a significant role in the prognosis of CRC patients as an independent risk factor for prognosis (34–37). However, CEA was not statistically meaningful for CSS while performing multi-logistic regression in our study. Hence, we made a new nomogram including CEA (**Figure S2**), of which AUC [training 0.829 95% CI, 0.790–0.869, validation 0.843 95% CI 0.772–0.913] and calibration plots (**Figures S3C, D**) show no significant difference between the new nomogram and the former. That is to say, the prediction efficiency can not be greatly improved by taking CEA into account. The ROC of the new nomogram is shown in **Figures S3A, B**.

During the study, we were puzzled by the differences between surgical and non-surgical patients (**Table S1**), which are mainly in the field of grade, T stage, and N stage. It was difficult to understand the fact that many patients with grade II, T1, or N0 staging were treated with no surgery, which is contrary to our previous perception. We believe that the reason for these incredible differences is the changes in the treatment modality for CRLM patients. With the development of medical technology, more and more CRLM patients were identified to be able to obtain survival benefits in surgical treatment, and surgery becomes the first choice for patients’ treatment. In our study, some people who lived in the time when the benefit of surgical treatment was not recognized were included. Although their condition was considered to meet the criteria for surgery now, they were not suggested to receive the surgery.

Compared with the nomogram currently published about predicting the prognosis of CRLM patients (5), our model performs better in terms of the accuracy of the nomogram.

What is more, CRLM patients diagnosed between 2004 and 2015 from the SEER database were collected for model construction and internal validation. An independent dataset was obtained from China for external validation. As a well-known database, the SEER database has larger and multi-center data compared with the limited data of our own center, which can improve the model’s predictive performance. However, these are two datasets covering highly different epidemiological, genetic, molecular, and cultural backgrounds, which are not free from potential selection bias.

However, there are still several limitations in the present study. Firstly, some factors reported to be significant for the prognosis of CRLM patients such as the number and size of liver metastasis were not investigated in this study because of the lack of relevant information (12, 15). Despite the lack of that information, our model still demonstrates a better performance for identifying CRLM patients than the TNM stage system, which encouraged us to continue this study. In the future, we are going to collect multi-center data to develop a modified CRLM (m-CRLM) nomogram that takes other significant indicators such as the size and number of liver metastases into account, in order to make our predicting tool more comprehensive and reliable. Secondly, the median CSS time is unable to reflect the overall survival characteristic of the non-surgical group, and some reference standards that could represent the prognostic status of the unoperated patient comprehensively are worthy of further research. Thirdly, due to the relatively limited amount of validation set, the performance of this model is still needed to be confirmed in a larger and prospective cohort.

## Conclusion

In summary, we have provided a novel and simple model to identify stage M1a CRLM patients who could indeed benefit from surgery. This predicting model could output individualized results with good accuracy, availability, and applicability. This nomogram might influence the clinician’s decision making in the process of treatment.

## Data availability statement

Publicly available datasets were analyzed in this study. This data can be found here: https://seer.cancer.gov/.

## Ethics statement

Ethical review and approval was not required for the study on human participants in accordance with the local legislation and institutional requirements. Written informed consent for participation was not required for this study in accordance with the national legislation and the institutional requirements.

## Author contributions

XJ and YW designed the study. XJ, YW, YF, and ZL collected the data and analyzed the data. XJ and YW drafted the manuscript. TZ, BY, and NZ reviewed the manuscript. WZ and LW were responsible for the whole project and supervised the study. All authors contributed to the article and approved the submitted version.

## Funding

This work was funded by the National Natural Science Foundation of China (81874056, 81874182), Shanghai Natural Science Foundation Project (22ZR1413300), the National Key Project of China (2017ZX10203204-007-004), the Public Health Bureau Foundation of Shanghai (201840019, 201940043), and 2019 clinical science and technology innovation project (SHDC12019X19).

## Conflict of interest

The authors declare that the research was conducted in the absence of any commercial or financial relationships that could be construed as a potential conflict of interest.

## Publisher’s note

All claims expressed in this article are solely those of the authors and do not necessarily represent those of their affiliated organizations, or those of the publisher, the editors and the reviewers. Any product that may be evaluated in this article, or claim that may be made by its manufacturer, is not guaranteed or endorsed by the publisher.

## Supplementary material

Supplementary Figure 1 Kaplan-Meier plot of CSS in stage M1a CRLM patients according to primary and metastatic resection. CSS, cancer specific survival; CRLM, colorectal cancer with liver metastasis.

Supplementary Figure 2 Construction of the new CRLM nomogram with the addition of CEA. CRLM, colorectal cancer with liver metastasis; CEA, carcinoembryonic antigen.

Supplementary Figure 3 Validation of the new CRLM nomogram. (A, B) ROC curve for discrimination in the training and validation cohorts. (C, D) Calibration plots for the actual (observed) and predicted probabilities of the new CRLM nomograms in the training and validation cohorts.

Supplementary Figure 4 Decision curve analysis of new CRLM nomogram with the addition of CEA in the training and validation cohorts. Horizontal lines indicates that no cases will experience the event; Gray lines indicates that all cases will experience the event; Red and blue lines represent the net benefits across threshold probabilities according to the CRLM nomogram and TNM classification, respectively. CRLM, colorectal cancer with liver metastasis; TNM, tumor, node, and metastasis; CEA, carcinoembryonic antigen.

Supplementary Figure 5 External validation of the nomogram. (A) ROC curve for discrimination in the FUSCC cohorts. (B) Calibration plots for the actual (observed) and predicted probabilities of the nomogram in the FUSCC cohorts. (C) Decision curve analysis of CRLM nomogram in the FUSCC cohorts. ROC, receiver operating characteristic curve; FUSCC, Shanghai Cancer Center, Fudan University.

Supplementary Table 1 Characteristics of M1a CRLM patients.

Supplementary Table 2 Characteristics of training set, internal and external validation set.
