# Supplementary material for: A population-based predictive model identifying optimal candidates for primary and metastasis resection in patients with colorectal cancer with liver metastatic
Source: Front Oncol. 2022 Oct 7;12:899659. doi: 10.3389/fonc.2022.899659 (PMC9585382; doi:10.3389/fonc.2022.899659)
Supplement: Supplementary Figure 1 — Kaplan-Meier plot of CSS in stage M1a CRLM patients according to primary and metastatic resection. CSS, cancer specific survival; CRLM, colorectal cancer with liver metastasis. [file DataSheet_1.zip › supplementary files/Table S1.docx]

**Table S1.** Characteristics of M1a CRLM patients.

| Parameters | All CRLM Patients  n=1,220(%) | Non-surgery  n=339(%) | Surgery  n=881(%) | *P value* |
| --- | --- | --- | --- | --- |
| Age |  |  |  | <0.001 |
| ＜50 | 253 (20.7) | 39 (11.5) | 214 (24.3) |  |
| 50≤X＜70 | 672 (55.1) | 189 (55.8) | 483 (54.8) |  |
| ≥70 | 295 (24.2) | 111 (32.7) | 184 (20.9) |  |
| Sex |  |  |  | 0.691 |
| Female | 569 (46.6) | 155 (45.7) | 414 (47.0) |  |
| Male | 651 (53.4) | 184 (54.3) | 467 (53.0) |  |
| Size |  |  |  | 0.343 |
| ≤3cm | 197 (16.2) | 51 (15.0) | 146 (16.6) |  |
| 3＜X≤5cm | 513 (42.0) | 137 (40.4) | 376 (42.7) |  |
| 5＜X≤7cm | 313 (25.6) | 86 (25.4) | 227 (25.7) |  |
| ＞7cm | 197 (16.2) | 65 (19.2) | 132 (15.0) |  |
| Race |  |  |  | 0.143 |
| Black | 204 (16.7) | 69 (20.3) | 135 (15.3) |  |
| White | 896 (73.4) | 242 (71.4) | 654 (74.3) |  |
| Other | 119 (9.8) | 28 (8.3) | 91 (10.3) |  |
| Unknown | 1 (0.1) | 0 (0.0) | 1 (0.1) |  |
| Grade |  |  |  | <0.001 |
| I | 37 (3.0) | 11 (3.2) | 26 (3.0) |  |
| II | 807 (66.2) | 161 (47.5) | 646 (73.3) |  |
| III | 182 (14.9) | 42 (12.4) | 140 (15.9) |  |
| IV | 40 (3.3) | 2 (0.6) | 38 (4.3) |  |
| Unknown | 154 (12.6) | 123 (36.3) | 31 (3.5) |  |
| Histology |  |  |  | 0.012 |
| Adenocarcinoma | 1061 (87.0) | 308 (90.9) | 753 (85.5) |  |
| Other | 159 (13.0) | 31 (9.1) | 128 (14.5) |  |
| T stage |  |  |  | <0.001 |
| T1 | 191 (15.6) | 171 (50.4) | 20 (2.3) |  |
| T2 | 41 (3.4) | 7 (2.1) | 34 (3.8) |  |
| T3 | 662 (54.3) | 80 (23.6) | 582 (66.1) |  |
| T4 | 326 (26.7) | 81 (23.9) | 245 (27.8) |  |
| N stage |  |  |  | <0.001 |
| N0 | 381 (31.2) | 214 (63.1) | 167 (18.9) |  |
| N1 | 500 (41.0) | 110 (32.5) | 390 (44.3) |  |
| N2 | 339 (27.8) | 15 (4.4) | 324 (36.8) |  |
| Neoadjuvant Chemotherapy |  |  |  | <0.001 |
| No | 228 (18.7) | 102 (30.1) | 126 (14.3) |  |
| Yes | 992 (81.3) | 237 (69.9) | 755 (85.7) |  |
| Marital status |  |  |  | <0.001 |
| Married | 689 (56.5) | 161 (47.5) | 528 (59.9) |  |
| Separated or divorced | 134 (11.0) | 49 (14.5) | 85 (9.6) |  |
| Single | 234 (19.2) | 65 (19.2) | 169 (19.2) |  |
| Widowed | 120 (9.8) | 48 (14.1) | 72 (8.2) |  |
| Unknown | 43 (3.5) | 16 (4.7) | 27 (3.1) |  |
| CEA |  |  |  | <0.001 |
| Negative/normal | 237 (19.4) | 28 (8.3) | 209 (23.7) |  |
| Positive/elevated | 983 (80.6) | 311 (91.7) | 672 (76.3) |  |
| Primary tumor position |  |  |  | 0.011 |
| Left colon | 520 (42.6) | 135 (39.8) | 385 (43.7) |  |
| Right colon | 516 (42.3) | 136 (40.1) | 380 (43.1) |  |
| Rectum | 184 (15.1) | 68 (20.1) | 116 (13.2) |  |

*Abbreviation: CRLM, colorectal cancer with liver metastasis; CEA, carcinoembryonic antigen; P＜0.05 means the result is statistically significant.
