# Supplementary material for: A population-based predictive model identifying optimal candidates for primary and metastasis resection in patients with colorectal cancer with liver metastatic
Source: Front Oncol. 2022 Oct 7;12:899659. doi: 10.3389/fonc.2022.899659 (PMC9585382; doi:10.3389/fonc.2022.899659)
Supplement: Supplementary Figure 1 — Kaplan-Meier plot of CSS in stage M1a CRLM patients according to primary and metastatic resection. CSS, cancer specific survival; CRLM, colorectal cancer with liver metastasis. [file DataSheet_1.zip › supplementary files/Table S2.docx]

**Table S2.** Characteristics of training set, internal and external validation set.

| Parameters | Training set n=705(%) | Internal validation set n=176(%) | External validation set n=169(%) | *P value* |
| --- | --- | --- | --- | --- |
| Age |  |  |  | 0.009 |
| ＜50 | 170(24.1) | 44(25.0) | 37(21.9) |  |
| 50≤X＜70 | 387(54.9) | 96(54.6) | 115(68.1) |  |
| ≥70 | 148(21.0) | 36(20.4) | 17(10.0) |  |
| Sex |  |  |  | 0.005 |
| Female | 324(46.0) | 90(51.1) | 58(34.3) |  |
| Male | 381(54.0) | 86(48.9) | 111(65.7) |  |
| Size |  |  |  | <0.001 |
| ≤3cm | 121(17.2) | 25(14.2) | 70(41.4) |  |
| 3＜X≤5cm | 306(43.4) | 70(39.8) | 78(46.2) |  |
| 5＜X≤7cm | 184(26.1) | 43(24.4) | 14(8.3) |  |
| ＞7cm | 94(13.3) | 38(21.6) | 7(4.1) |  |
| Race |  |  |  | <0.001 |
| Black | 104(14.8) | 31(17.6) | 0(0) |  |
| White | 531(75.3) | 123(69.9) | 0(0) |  |
| Other | 70(9.9) | 21(11.9) | 169(100.0) |  |
| Unkown | 0(0) | 1(0.6) | 0(0) |  |
| Grade |  |  |  | <0.001 |
| I | 22(3.1) | 4(2.3) | 33(19.5) |  |
| II | 522(74.0) | 124(70.5) | 132(78.1) |  |
| III | 103(14.6) | 37(21.0) | 4(2.4) |  |
| IV | 31(4.4) | 7(4.0) | 0(0) |  |
| Unkown | 27(3.9) | 4(2.2) | 0(0) |  |
| Histology |  |  |  | 0.031 |
| Adenocarcinoma | 598(84.8) | 155(88.1) | 156(92.3) |  |
| Other | 107(15.2) | 21(11.9) | 13(7.7) |  |
| T stage |  |  |  | <0.001 |
| T1 | 12(1.7) | 8(4.5) | 4(2.4) |  |
| T2 | 31(4.4) | 3(1.7) | 21(12.4) |  |
| T3 | 468(66.4) | 114(64.8) | 104(61.5) |  |
| T4 | 194(27.5) | 51(29.0) | 40(23.7) |  |
| N stage |  |  |  | <0.001 |
| N0 | 147(20.9) | 20(11.4) | 68(40.2) |  |
| N1 | 307(43.5) | 83(47.1) | 65(38.5) |  |
| N2 | 251(35.6) | 73(41.5) | 36(21.3) |  |
| Neoadjuvant chemotherapy |  |  |  | <0.001 |
| No | 90(12.8) | 36(20.4) | 117(69.2) |  |
| Yes | 615(87.2) | 140(79.6) | 52(30.8) |  |
| Marital status |  |  |  | <0.001 |
| Married | 424(60.1) | 104(59.1) |  |  |
| Separated or divorced | 66(9.4) | 19(10.8) |  |  |
| Single | 138(19.6) | 31(17.6) |  |  |
| Widowed | 60(8.5) | 12(6.8) |  |  |
| Unknown | 17(2.4) | 10(5.7) |  |  |
| CEA |  |  |  | 0.002 |
| Negative/normal | 166(23.5) | 43(24.4) | 62(36.7) |  |
| Positive/elevated | 539(76.5) | 133(75.6) | 107(63.3) |  |
| Primary tumor position |  |  |  | <0.001 |
| Left colon | 308(43.7) | 77(43.7) | 59(34.9) |  |
| Right colon | 300(42.5) | 80(45.5) | 41(24.3) |  |
| Rectum | 97(13.8) | 19(10.8) | 69(40.8) |  |

CRLM, colorectal cancer with liver metastasis; CEA, carcinoembryonic antigen; P＜0.05 means the result is statically significant.
