# Supplementary figures and images for: A population-based predictive model identifying optimal candidates for primary and metastasis resection in patients with colorectal cancer with liver metastatic
Source: Front Oncol. 2022 Oct 7;12:899659. doi: 10.3389/fonc.2022.899659 (PMC9585382; doi:10.3389/fonc.2022.899659)

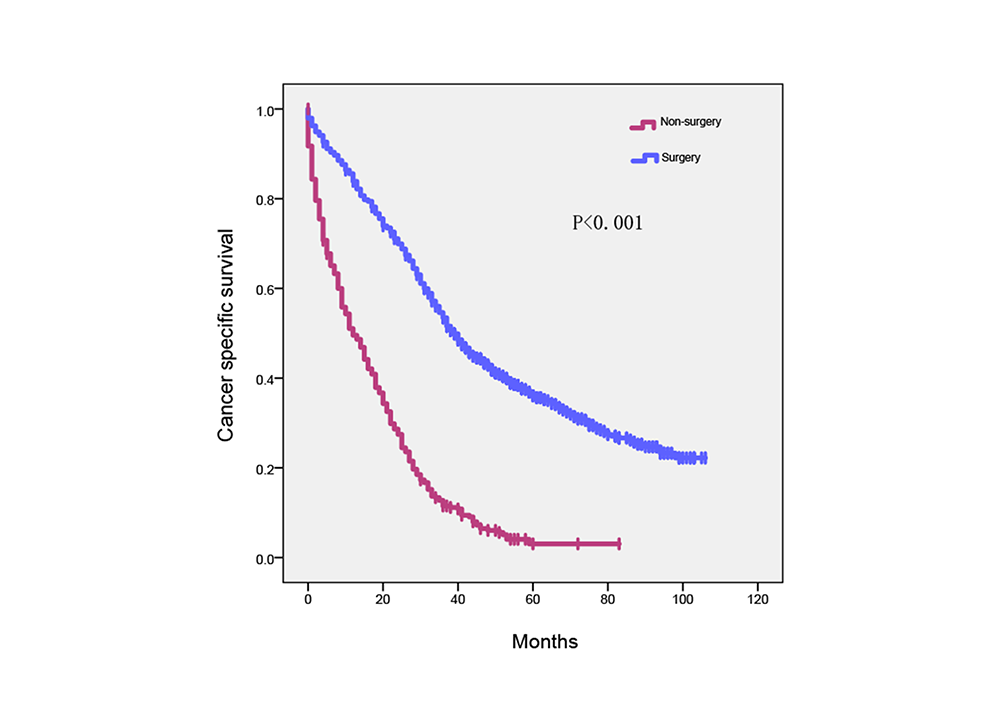

Supplement: Supplementary Figure 1 — Kaplan-Meier plot of CSS in stage M1a CRLM patients according to primary and metastatic resection. CSS, cancer specific survival; CRLM, colorectal cancer with liver metastasis. [file DataSheet_1.zip › supplementary files/Figure S1.tif]

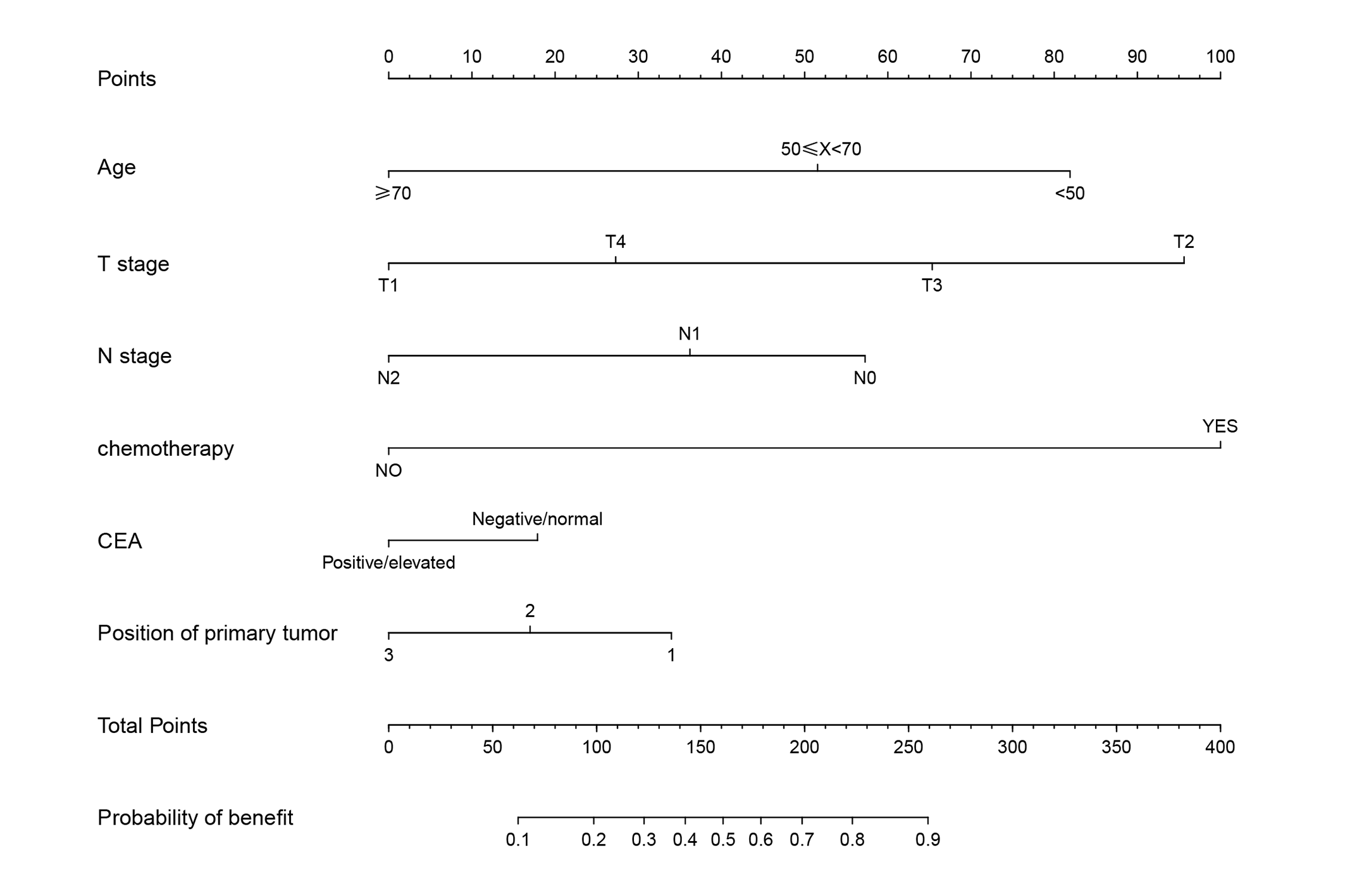

Supplement: Supplementary Figure 1 — Kaplan-Meier plot of CSS in stage M1a CRLM patients according to primary and metastatic resection. CSS, cancer specific survival; CRLM, colorectal cancer with liver metastasis. [file DataSheet_1.zip › supplementary files/Figure S2.tif]

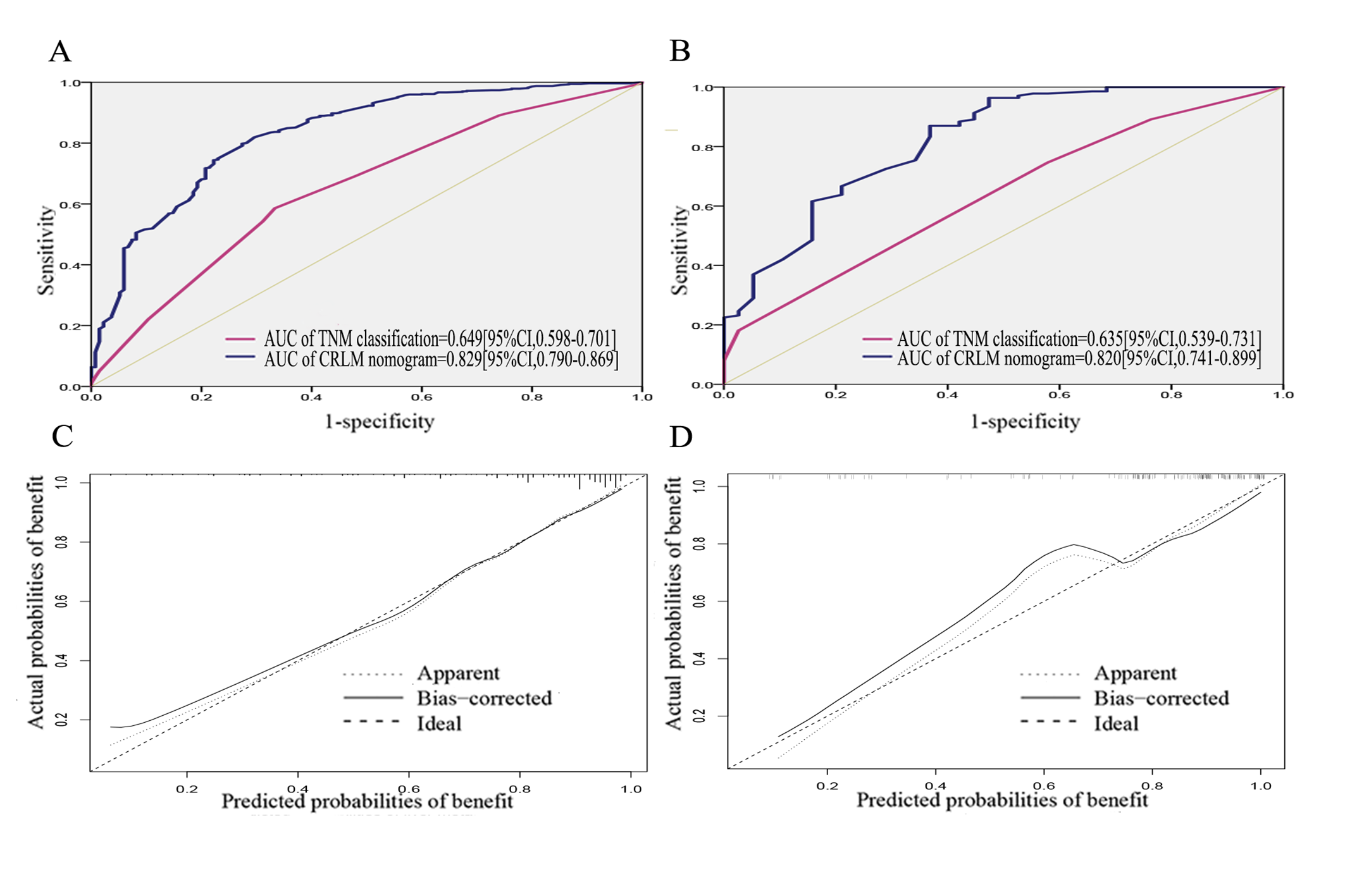

Supplement: Supplementary Figure 1 — Kaplan-Meier plot of CSS in stage M1a CRLM patients according to primary and metastatic resection. CSS, cancer specific survival; CRLM, colorectal cancer with liver metastasis. [file DataSheet_1.zip › supplementary files/Figure S3.tif]

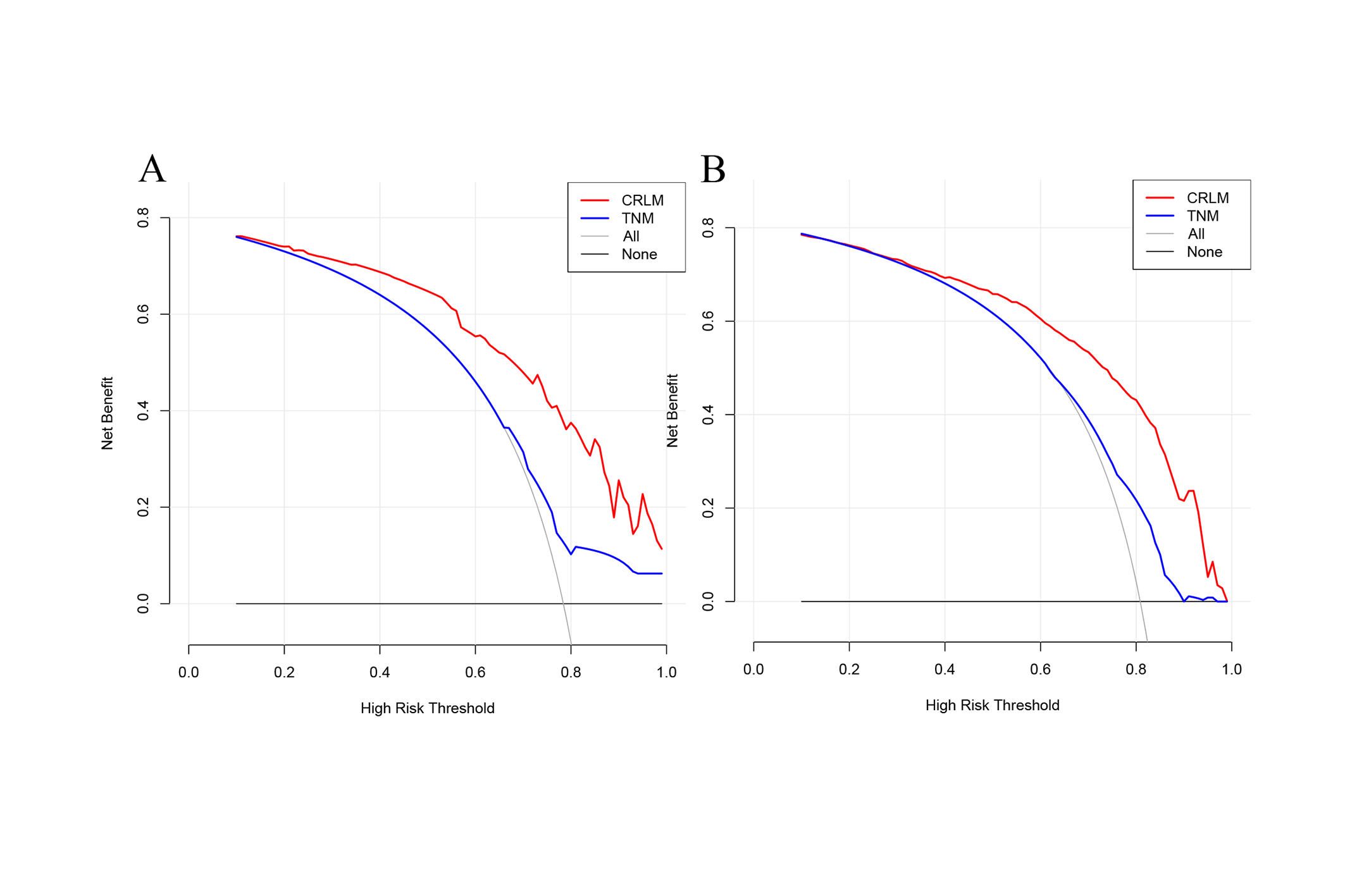

Supplement: Supplementary Figure 1 — Kaplan-Meier plot of CSS in stage M1a CRLM patients according to primary and metastatic resection. CSS, cancer specific survival; CRLM, colorectal cancer with liver metastasis. [file DataSheet_1.zip › supplementary files/Figure S4.tif]

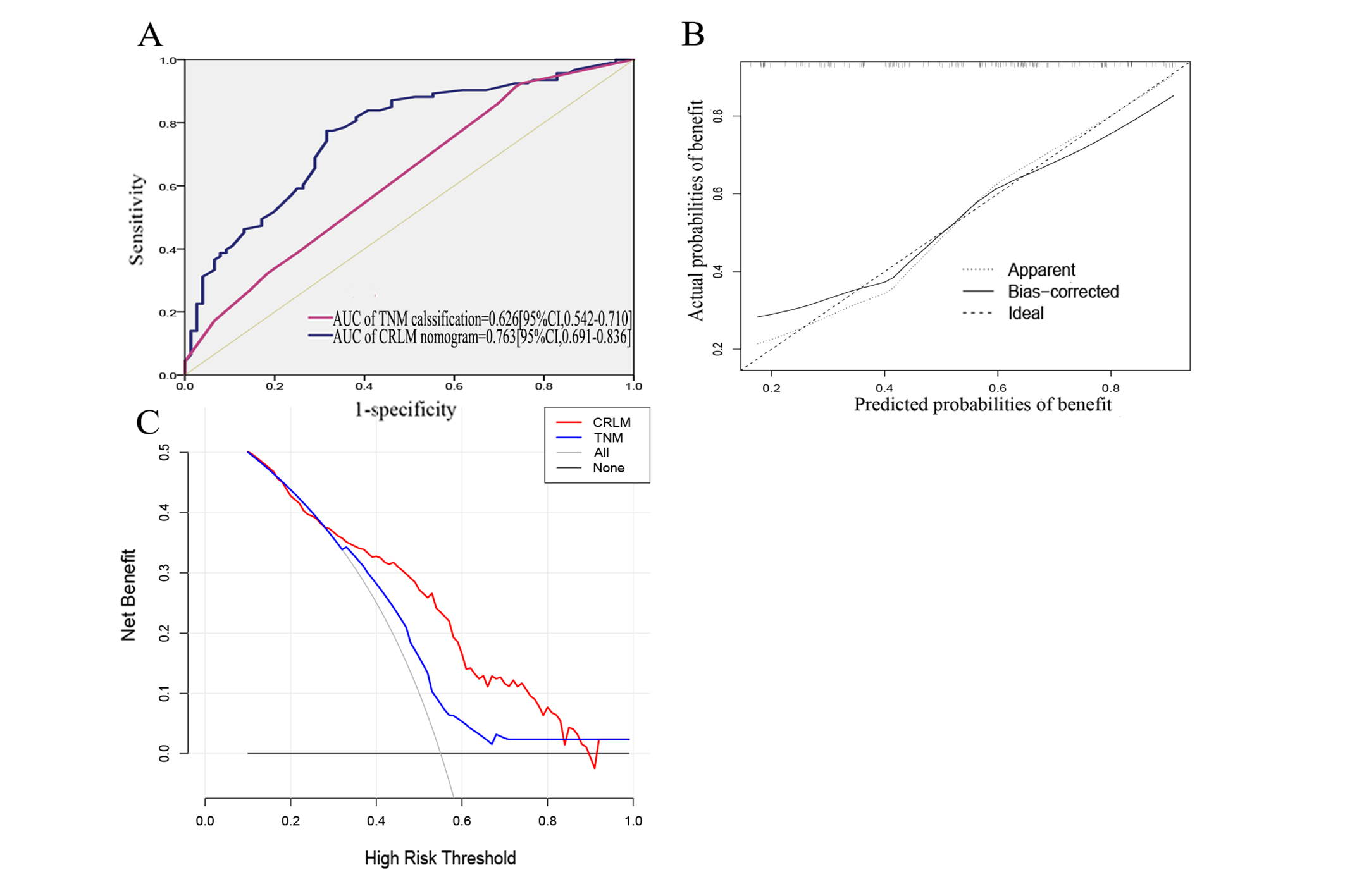

Supplement: Supplementary Figure 1 — Kaplan-Meier plot of CSS in stage M1a CRLM patients according to primary and metastatic resection. CSS, cancer specific survival; CRLM, colorectal cancer with liver metastasis. [file DataSheet_1.zip › supplementary files/Figure S5.tif]
